# Medicinal plants used by the Yi ethnic group: a case study in central Yunnan

**DOI:** 10.1186/1746-4269-5-13

**Published:** 2009-04-23

**Authors:** Chunlin Long, Sumei Li, Bo Long, Yana Shi, Benxi Liu

**Affiliations:** 1 Kunming Institute of Botany, Chinese Academy of Sciences, Kunming 650204, PR China; 2 College of Life and Environmental Sciences, Minzu University of China, Beijing 100081, PR China; 3 Graduate School, Chinese Academy of Sciences, Beijing 100039, PR China; 4 College of Horticulture, Yunnan Agricultural University, Kunming 650201, PR China; 5 Chuxiong Research Institute of Yi Medicine and Pharmacology, Yunnan 671000, PR China; 6 South China Sea Institute of Oceanology, Chinese Academy of Sciences, Guangzhou 510301, PR China

## Abstract

**Background:**

This paper is based on ethnomedicinal investigation conducted from 1999–2002 in Chuxiong, central Yunnan Province, Southwest China. The Yi medicine has made a great contribution to the ethnomedicinal field in China. Neither case studies nor integrated inventories have previously been conducted to investigate the traditional Yi plants. This paper aims to argue the status and features of medicinal plants used in traditional Yi societies through a case study.

**Methods:**

The approaches of ethnobotany, anthropology, and participatory rural appraisal were used in the field surveys. Twenty-two informants in four counties were interviewed during eight field trips. Medicinal plant specimens were identified according to taxonomic methods.

**Results:**

One hundred sixteen medicinal plant species were found to be useful by the local people in the treatment of various diseases or disorders, especially those relating to trauma, gastrointestinal disorders and the common cold. Among these 116 species, 25 species (21.55%) were found to have new curative effects and 40 species (34.48%) were recorded for their new preparation methods; 55 different species were used in treating wounds and fractures, and 47 were used to treat gastrointestinal disorders. Traditional Yi herbal medicines are characterized by their numerous quantities of herbaceous plants and their common preparation with alcohol.

**Conclusion:**

Totally 116 species in 58 families of medicinal plants traditionally used by the Yi people were inventoried and documented. The characteristics of medicinal plants were analyzed. Some new findings (such as new curative effects and new preparation methods) were recorded These newly gathered ethnobotanical and medicinal data are precious sources for the future development of new drugs, and for further phytochemical, pharmacological and clinical studies.

## Background

### Study Area

Chuxiong Prefecture, located in the north of middle Yunnan Plateau (24°13' – 26°30' N, 100°43' – 102°43' E), has numerous high mountains, deep canyons, and large and small rivers. Mountainous land in Chuxiong occupies over 90% of its total territory. Chuxiong's climate is considered a sub-tropical and moist plateau monsoon climate, primarily affected by two air currents: a warm, dry current from northern Africa, the Middle-East and the subcontinent of India during winter and a cool, moist current from the Indian Ocean and Bay of Bengal during summer. The climate of Chuxiong is characterized by long spring and autumn and short summer and winter. Although the daily temperature can vary greatly, the climate is generally temperate and without extremes [1]. The Ailao Mountains contain large forests and are abundant with medicinal plants. The Yi have utilized these plants in the prevention and cure of disease for centuries. Some 871 species of Yi plant medicines have currently been recorded [2].

### Population

With the exception of the Han Chinese, there are 55 minority nationalities in China. Each of them has their own unique history and medicinal system. With a population of 6.57 million, the Yi are the sixth largest minority in China, distributed in Yunnan, Sichuan, Guizhou and Guangxi provinces. In the southwest of China, Yunnan holds the largest population of the Yi nationality [1], which reaches 4.06 million.

### Aim of study

Research into the literature reveals that most of the ancient books on Yi medicine were handed down by dictation and are hand-written. Therefore, there must be additional traditional knowledge on Yi medicine that was scattered among folk areas and unrecorded, including some proven remedies with special curative effects preserved by folk doctors [3]. We thus decided to collect the folk knowledge of Yi medicine in Chuxiong Prefecture to compliment the previously documented works on traditional Yi medicine.

### Previous knowledge on local folk medicine

The Yi people mainly inhabit mountainous areas or river valleys. The particular geology and climate is ideal for a unique Yi medicine effective in treating pyretic toxicity, rheumatics, etc. Yi medicine, practiced for 3000 years, is derived from Dali medicine of the Nanshao Kingdom. By assimilating the best of local, Indian, and Persian medicine [4], the Yi people built their own medicinal theoretical system. The Yi acquired extensive clinical experience and recorded the pertinent knowledge in books, such as *Xian Yao Jing*, *Shuangbai Medicinal Book of Yi Nationality*, *Book for Curing Diseases*, and others. The earliest book of Yi medicine is *The Shuangbai Medicinal Book of Yi Nationality*, written even earlier than Shi-Zhen Li's *Ben Cao Gang Mu *in 1758. As we know, traditional medicine (TM) is widely used and is of increasing importance in a rapidly growing global health and economic system. In Africa, up to 80% of the population uses TM to help to meet their health care needs. In Asia and South America, populations continue to use TM as a result of historical circumstances and cultural beliefs. In China, TM accounts for around 40% of all health care delivered [5]. Given these statistics, we should cherish, protect and develop upon the legacy that is traditional Yi medicine.

The majority of products which were exploited in recent years based on Yi medicines have provided positive social and financial benefits. The famous Yi doctor, Huan-Zhang Qu, researched and developed the outstanding *Yunnan Bai Medicine *[6]. Similar drugs such as the *Capsule of Paiduyangyan*, the *Kunming Shanhaitang*, the *Injection of Yunnan Dengzhanhua*, *Injection of Sanqi saponin*, *Capsule of Yixinkang *and others have all been developed following deep research into the culture of traditional Yi medicine. The selling price of these drugs derived from traditional Yi medicines could reach 10 billion Chinese Yuan (or 1.26 billions in US dollars) per year [6].

Indigenous knowledge, and folk knowledge in particular, continues to be impacted by mainstream culture and decreasing biodiversity; traditional practices using specific medicinal plants decreases more quickly than that of the general biodiversity. It is thus urgent and necessary to prevent the further loss of the specialized knowledge of minority peoples. This is best accomplished by gathering and documenting their unique practices and their relationships to medicinal plants [6].

## Methodology

Eight field surveys in four counties were carried out during different seasons over a period of four years (1999–2002). These four locations in Chuxiong Prefecture consisted of: Wuding, Shuangbai and Nanhua counties, and Chuxiong City. The methodological approaches were used in ethnobotany, anthropology, and participatory rural appraisal (PRA) [7-10] were adopted for the field investigations.

During each visit, detailed field records were taken on the functions of plants and the plant parts that were used. Information was obtained through interviews with 32 informants including elderly villagers, local healers, and herbalists. The authors collected voucher specimens of 116 plants in the field with assistance from local herbalists. The specimens were later verified using *The Flora of China *and *Illustrated Handbook for Higher Plants of China *[11,12], and by the authors, plant taxonomists, and experts from the authors' institution. The voucher specimens were deposited in the Voucher Herbarium of the Laboratory of Ethnobotany, Kunming Institute of Botany, Chinese Academy of Sciences. The local names of the herbs, their life forms, preparation methods, and their useful parts for treating various diseases were carefully recorded in the field [13]. The efficacy of these plants was also analyzed and compared with the pertinent literature.

## Results

### Statistics of plants used

One hundred and sixteen plant species used for medicinal purposes by people living in Chuxiong Prefecture of Yunnan Province were collected and identified (Additional file 1). These species are represented by 58 different families, of which Compositae (15 species), Rosaceae (6 species), and Liliaceae, Papilionaceae and Orchidaceae (each with 5 species) hold the highest number of species used medicinally. One hundred and four genera are represented: *Ainsliaea*, *Artemisia*, *Clematis*, *Clerodendrum*, *Conyza*, *Corydalis*, *Cynanchum*, *Indigofera*, *Pholidota*, *Polygala*, *Polygonum *and *Rubus *all occurring twice.

25 previously unidentified species (21.55%) were found to have new curative effects and 40 species (34.48%) had preparation methods that have never been recorded in the contemporary medical texts of the Yi nationality in Chuxiong Prefecture (Additional file 1) [14-17].

### Medicinal uses

The three most common conditions treated with Yi plants were: trauma, such as wounds and fractures, for which 55 different species were used, eg. *Crepis napifera *and *Gaultheria yunnanensis*; gastrointestinal disorders, such as stomachache, diarrhea, and constipation; and the common cold, such as fever, cough, sore throat and influenza (for example, *Murraya paniculata *and *Polygonum cymosum*) (Table 1). Only nine species were reported as being useful against a single disease, eg. *Ranunculus ternatus *and *Euonymus japonicus*.

**Table 1:** List of the frequency of ailments treated with medicinal plants

Disease	Species used	% of total
Trauma	55	47.41%
Gastrointestinal disorders	47	40.52%
Common cold	24	20.69%
Skin problems	20	17.24%
Liver problems	19	16.38%
Gynaecopathia	18	15.52%
Inflammation	13	11.21%
Trachea/bronchi disorder	12	10.34%
Edema	11	9.48%
Lung problems	11	9.48%
Antitoxics	10	8.62%
Heart problems	10	8.62%
Body pains	9	7.76%
Female issues	5	4.31%
Blood problems	4	3.45%
Eye problems	4	3.45%
Kidney problems	4	3.45%
Neural diseases	3	2.59%
Cypridopathy	2	1.72%
Ear problems	2	1.72%
Mouth cavity problems	2	1.72%
Infectious disease	1	0.86%
Parasitic diseases	1	0.86%
Rhachitis	1	0.86%
Tooth problems	1	0.86%

### Plant habit and part used

Seventy two plants, over half of the species used medicinally, were herbaceous in nature (eg. *Ainsliaea yunnanensis *and *Cucubalus baccifer*), while 33 were woody, i.e. tree, shrub, or woody liana (eg. *Aquilaria sinensis *and *Toona sinensis*), and 11 were sub-shrubs. The entire plant was used most often, accounting for 65 species out of 116 (56.03%), followed by the roots, of 42 species out of 116 (36.21%); other plant parts were used less frequently, about 7.76%.

### Preparing methods of medicinal plants

As shown in Additional file 1, the preparing methods of medicinal plants were different and special. Eighty-eight species could be taken by decoction, which was the most common preparing method. Seventeen species should be used after they were soaking in alcohol. Four species could be used after making juice for drinking or external uses. Fourteen species could be used by making powder. Some species should be used together with other species, or with sugar, honey, meat, eggs, chicken, or other materials.

## Conclusion

In the present study, 116 species belonging to 58 families have been identified as medicinal plants used by Yi healers in Chuxiong Prefecture, Yunnan Province. These plants have unique properties and are used in the treatment of trauma, gastrointestinal disorders and the common cold. From the statistic data, we found most of the plants are used to treat trauma. This is because the primitive ancestors of the Yi people usually lived in tree crotches and made their living by hunting and gathering [6], which made them vulnerable to injury. The medicinal plants used to treat gastrointestinal disorders accounts for the second largest percentage. This result can be explained by the epidemiological research on *Helicobacter pylori *(Hp) among Yi communities. The Yi people were easily infected by Hp, which has a strong correlation with their fondness for drinking distilled spirits, smoking, and eating pickled, fermented, and fried food [18].

There are several characteristics of traditional Yi medicine. Most plants used in Yi medicine are herbaceous as shown in Additional file 1. Among 116 medicinal plant species traditionally used by the Yi healers in the case sites, 72 species are either perennial or annual herbs, or herbaceous lianas. This is partially due to the Yi's polytheistic beliefs. The Yi believe that both mountains and big trees are sacred and should not be harmed [19]. This perception of nature plays a positive role on the protection of the vegetation around their dwelling places. The minorities who live in mountainous areas would never intentionally damage their natural environment and aim to exist in harmony with it. That is why many scholars advocate that people living in modern society learn from the minority people, to respect the environment rather than depredating it [20-25].

Fresh herbs are used almost exclusively by Yi healers. Dry herbs are used, but much less frequently. The Yi healers usually used a single herb instead of multi-species compounds. For example, when the Yi healer treated wounds with *Gaultheria yunnanensis*, he would not mix it with any of the other (over 50) species he knew that could also treat wounds. Some healers were less concerned about measuring doses when administering medicinal herbs. Medicinal plant preparation methods vary: For external application, plants are generally pounded, kneaded or chewed; for oral doses, plants are often chewed, decocted or cooked with meat.

Traditional Yi medicine is especially characterized by its use of alcohol. The Yi people adore alcohol, and it has come to symbolically represent this cultural minority due to its significance in their lives. Home-made alcohol is the most important beverage for the Yi: it is used daily, for ceremonies and holidays, served to respected guests and friends, and is the most common method of administering plant medicines. The practice of combining plants and alcohol has a long history in Yi medicine. Yi healers use different procedures to administer their raw material/alcohol combinations. They most commonly use: 1) medicinal alcohol: medicinal plants and/or animals are soaked in alcohol for about a month and the resulting liquid then is drunk by the patient or applied externally to the affected parts; 2) alcohol decoction: herbs are put into alcohol, decocted and drunk; 3) alcohol as solvent instead of water: fresh plant liquid or dried plant powder is placed in alcohol and either drunk or applied externally; and 4) burning alcohol in medicine: medicinal plants are mixed with alcohol, heated, and then either taken by mouth or applied externally. It is believed that alcohol extracts more active components from the medicinal plants than water does, thus being more effective in curing diseases. For example, *Ainsliaea latifolia *var. *obovata*, when soaked in alcohol, is far more effective in treating children's fever than the fresh plant alone. This traditional knowledge was previously unreported.

Since natural medicines can cure diseases ranging from bacterial infections to cancers, pharmaceutical companies and the public recognize the importance of its development. Most of the pharmaceutical companies believe that the biggest benefit hiding behind the traditional plant knowledge and practices of minority people is the value of new drug development [2].

Biodiversity is a rare gift provided by nature to those researching chemo-diversity and the discovery of new drugs. This chemo-diversity not only provides the necessary secondary metabolites of plant evolution, but also provides abundant lead compounds, the basis of new drug development [2]. Due to the side-effects associated with chemosynthetic medicine and environmental pollution, medical scientists from both here and abroad have begun to look into the realm of traditional medicine, which has used natural medicine as its main remedial measure [26]. During our research, we found twenty five species with previously undocumented curative effects and forty species whose preparation methods and compatibilities had not been documented. These new records can be an important source for further phytochemical, pharmacological and clinical studies.

We suggest that the traditional knowledge of the Yi could provide useful information in finding new drugs that contribute to human welfare.

## Competing interests

The authors declare that they have no competing interests.

## Authors' contributions

Author C Long conducted field surveys and interviews with the healers, identified the herbarium specimens, drafted and finalized the manuscript with S Li and Y Shi. Author B Long provided assistance when preparing the manuscript. Author B Liu helped to analyze the data.

## Supplementary Material

Additional file 1 **Inventory of Traditional Herbal Plants Used by the Yi People**. One hundred and sixteen plant species used for medicinal purposes by people living in Chuxiong Prefecture of Yunnan Province. Click here for file
