# Supplementary material for: Medicinal plants used by the Yi ethnic group: a case study in central Yunnan
Source: J Ethnobiol Ethnomed. 2009 Apr 23;5:13. doi: 10.1186/1746-4269-5-13 (PMC2679000; doi:10.1186/1746-4269-5-13)
Supplement: Additional file 1 — Inventory of Traditional Herbal Plants Used by the Yi People. One hundred and sixteen plant species used for medicinal purposes by people living in Chuxiong Prefecture of Yunnan Province. [file 1746-4269-5-13-S1.pdf]

Inventory of Traditional Herbal Plants Used by the Yi People

Scientific Name	Family Name	Local Name	Life Form	Parts Used	Medicinal Uses	Preparing Methods
Acanthopanax trifoliatus Merr.	Araliaceae	baoqiluosai	Shrub	Root	Rheumatoid arthritis, jaundice, wound, fever	New: decoction with (without) radish piece
Achyranthes aspera L.	Amaranthaceae	ninajiezai	Annual or biennial herb	Root	Irregular menstruation, gonorrhea	Decoction
Aesculus chinensis Bunge.	Hippocastanaceae	peizou	Deciduous arbor	Bark	Stomachache, toothache	New: decoction with (without) *Achyranthes aspera* L.
Agapetes lacei Craib	Ericaceae	faluoxi	Evergreen shrub	Root	Wound, stomachache, hepatitis, edema	Decoction with (without) *Foeniculum vulgare* roots
Agrimonia zeylanica Moon.	Rosaceae	eshiyema	Perennial herb	Whole plant	Migraine, blood problem	Decoction with (without) *Caesalpinia sappan*
Ainsliaea latifolia var. *obovata* Grier. et Lauener.	Compositae	peibuluo	Perennial herb	Whole plant	New: high fever (children)	New: soaking in alcohol
Ainsliaea yunnanensis Franch.	Compositae	ruoluwa	Perennial herb	Whole plant	Joint problems, wound, fracture New: madness	Decoction with alcohol
Ajuga decumbens Thunb.	Labiatae	wubonuo	Perennial herb	Whole plant	Trachitis, wound, madness, gonorrhea	Making juice for external using; New: decoction with (without) sugar
Albizzia kalkora Prain.	Mimosaceae	qiaosaimilu	Deciduous arbor	Flower	Rheumatic arthritis, fracture, sleeplessness	New: decoction with (without) *Foeniculum vulgare* roots
Allium tuberosum Roxb.	Liliaceae	ci'abo	Perennial herb	Root, leaf	Wound, dysentery, regurgitate	Making juice and drinking or for external using
Ampelopsis delavayana Planch.	Vitaceae	yaonübei	Deciduous climbing	Root	Wound, fracture, rheumatic arthritis	Decoction with (without) *Tetrastigma hypoglaucum*
Anemone rivularis Buch-Ham.	Ranunculaceae	aimulieshi	Perennial herb	Whole plant	Amygdalitis, chronic hepatitis, stomachache, swelling	Soaking in alcohol; New: making juice or power
Anisodus acutangulus C.Y. Wu et C. Chen	Solanaceae	lejuecai	Perennial herb	Root, leaf	New: stomachache, wound, fracture	New: decoction

Apium graveolens L.var. *dulce* DC.	Umbelliferae	nedaimu	Perennial cultivated herb	Whole plant	Wind syndrome of head	New: decoction; or making power with *Allium tuberosum*
Aquilaria sinensis Gilg.	Thymelaeaceae	liwaziwa	Evergreen arbor	Bark	Emesia, asthma;<br>New: constipation	Making powder and drinking;<br>New: decoction with *Amomum tsao-kuo*
Aristolochia calcicola C.Y. Wu	Aristolochiaceae	hannuonaci	Ligneous liana	Root	Hypertension, cold;<br>New: snakebite	New: making powder with *Heracleum lanatum*
Artemisia apiacea Hance	Compositae	shiwubai	Annual herb	Whole plant	Nephritis, cold, diarrhea, enteritis	Decoction with (without) *Laggera pterodonta*
Artemisia japonica Thunb.	Compositae	erkeyika	Perennial herb	Whole plant	Bacillary phthisis, Hypertension, mouth cavity problems	New: decoction with (without) *Myriactis delavayi*
Aspidistra elatior Blume	Liliaceae	sheluojie	Perennial evergreen	Whole plant	Cough, wound, fracture, bellyache	Decoction or soaking in alcohol
Bauhinia faberi Oliver.	Caesalpiniaceae	peimili	Little shrub	Root, leaf	Neurosis, wound	Decoction with roots (or roots and stems)
Begonia yunnanensis Levl.	Begoniaceae	baikaiheitang	Perennial herb	Whole plant	Irregular menstruation, stomachache, wound, emesia	New: decoction with soup of sour pickles
Bidens bipinnata L.	Compositae	nenshi	Annual herb	Whole plant	Dysentery, wound, fever	New: pounding into pulp for the external application, decoction with *Dendranthema*
Boehmeria nivea Gaud.	Urticaceae	jimabai	Perennial sub-fruticose	Root	Abortion, cold, edema caused by Nephritis, wound	Decoction with lotus and yam
Buddleia officinalis Maxim.	Loganiaceae	weizhongzen uo	Shrub	Root, leaf, flowers	Cough, eye problems; New: hepatitis, asthma,	New: decoction (making primer with alcohol) stewing leaf with eggs
Cacalia palmatisecta Hand.-Mazz.	Compositae	luohua	Perennial herb	Whole plant	Common cold, Cough, wound	Decoction
Campylotropis trigonoclada Schneid.	Papilionaceae	songlouzhen g	Little shrub	Root	Gonorrhea, edema, wound	New: decoction with *Ainsliaea latifolia* var. *obovata*
Carpesium cernuum L.	Compositae	nuobatijiedu o	Perennial herb	Whole plant	Cold, toothache, dysentery<br>New: rhachitis	New: decoction with (without) *Cynoglossum lanceolatum*
Cassia obtusifolia L.	Caesalpiniaceae	zandoujian	Annual sub-fruticose	Seed	Ceratitis, hypertension, stomachache, headache	Decoction (for external using)

Chenopodium ambrosioides L.	Chenopodiaceae	binise	Annual or perennial herb	Whole plant	Cold, ancylostomiasis, nematodiasis, eczema,	New: making pellets with sugar and *Setaria italica*
Chrysanthemum indicum L.	Compositae	chidou'oudou	Perennial herb	Whole plant	Cold, hypertension, gastritis and enteritis, eczema, anthracia	Decoction and drinking; making juice (for external using)
Clematis obtusidentata Hjeichler	Ranunculaceae	yuwuli	Liana	Whole plant	Cystitis, urethritis	Decoction
Clematis peterae Hand.-Mazz.	Ranunculaceae	nimohuangs hi	Perennial evergreen	Root, stem	Edema, palmus	Decoction with meat and *Adenophora bulleyana*
Clerodendrum bungei Steud.	Verbenaceae	xichiji	Deciduous shrub	Whole plant	Hernia, leukorrhea, hypertension, hemorrhoid	New: decoction with the roots of *Toona ciliata*
Clerodendrum petasites Moore	Verbenaceae	maiheiao	Shrub	Leaf	Wound, hepatitis, sore throat, bronchitis	Decoction with sugar
Conyza blinii Lévl.	Compositae	jiqiaoshi	Annual herb	Whole plant	Amygdalitis, nephritis, toothache, chronic bronchitis	Decoction with honey or brown sugar
Conyza japonica Less.	Compositae	heibainongsh i	Annual or biennial herb	Whole plant	Eczema, amygdalitis, laryngitis, alveolysis	Decoction<br>New: gargle with medicine water
Corydalis edulis Maxim.	Papaveraceae	benzhiduoqi	Annual herb	Root	Bacillary phthisis, sore throat, otitis media	Stewing with pig's knuckles
Corydalis thalictrifolia Franch.	Papaveraceae	wazimoci	Perennial herb	Whole plant	Hemorrhoid, stomachache	Decoction with alcohol; or making powder for external using
Crepis napifera Babc.	Compositae	taluwa	Perennial herb	Root	Stomachache, bronchitis, cough, wound	New: making powder after removed the bubble
Cryptolepis buchananii Roem. et Schult.	Asclepiadaceae	aqina	Liana	Root	Wound, fracture, stomachache	Decoction; or soaking in alcohol
Cucubalus baccifer L.	Caryophyllaceae	kahousi	Perennial herb	Whole plant	Wound, fracture, hernia, edema, bacillary phthisis	pounding into pulp for external using; or soaking in alcohol
Cuscuta chinensis Lam.	Cuscutaceae	menyishi	Annual autoecious	Whole plant	Leukorrhea, eczema, hepatitis	Decoction with honey or brown sugar
Cynanchum amplexicaule Hemsl.	Asclepiadaceae	nuopeinuoci	Perennial herb	Root	Eczema, enteritis, irregular menstruation	Making powder (taking in alcohol)<br>New: taking juice which squeeze roots with

Cynanchum otophyllum Schneid.	Asclepiadaceae	roujiboqi	Perennial herbal liana	Root	Wound, stomachache, madness, sonitus, epilepsia	Decoction with meat; or soaking in alcohol
Cynodon dactylon Pers.	Gramineae	momozalabai	Perennial herb	Whole plant	Wound, toothache, hepatitis;<br>New: dysentery	Pounding into pulp for external application<br>New: stewing with pork and *Adenophora*
Cynoglossum lanceolatum Forsk.	Boraginaceae	anudeniang	Herb	Root	Irregular menstruation, cystitis	Decoction
Dactylicapnos scandens Hutch.	Papaveraceae	wugeinü	Perennial herbal liana	Root	Pains, wound;<br>New: dysentery	Decoction or powder (internal use); or soaking in alcohol
Daphne feddei Lévl.	Thymelaeaceae	aluobaluoji	Evergreen shrub	Whole plant	Wound, rheumatoid arthritis, stomachache	Making pellet or powder, decoction with honey
Desmodium microphyllum DC.	Papilionaceae	mindexiong	Perennial creepy herb	Whole plant	Menstruation problems, dysentery	Decoction with (without) G*aultheria yunnanensis* and brown sugar
Dichrocephala benthamii C. B. Clarke.	Compositae	womidu	Annual herb	Whole plant	Stomachache, mouth problems	Decoction<br>New: squeeze juice with alcohol for
Didymocarpus yunnanensis C. E. C. Fisch.	Gesneriaceae	sinuoqi	Perennial herb	Whole plant	Wound, cough, fracture	Stewing with alcohol, making powder (for external using)
Diospyros mollifolia Rehd et Wils.	Ebenaceae	seyili	Evergreen shrub or little	Fruit, leaf	Chronic diarrhea, dyspepsia (child)	Decoction with *Crataegus pinnatifida*
Disporum cantoniensis Merr.	Orchidaceae	chenshan	Perennial herb	Root	Menstruation problem<br>New: bacillary phthisis	Decoction with eggs
Duchesnea indica Focke.	Rosaceae	shelici	Perennial herb	Whole plant	Irregular menstruation, snake bites	Pounding into pulp the external application<br>New: decoction with alcohol and brown
Eria pannea Lindl.	Orchidaceae	luoduo	Perennial adnascent	Whole plant	Wound, fracture, medicinal poisoning, chronic bronchitis	Decoction with big red ginseng
Eriosema chinense Vogel.	Papilionaceae	qubenjing	Herb	Root	Liver and Lung problems	Decoction with sugar or honey
Euonymus japonicus Thunb.	Celastraceae	houbaikeben shi	Evergreen shrub or ardor	Root	Irregular menstruation	Stewing with meat
Evodia lepta Merr.	Rutaceae	shaochaoshi ka	Deciduous shrub or little	Whole plant	Rheumatoid arthritis, skin problems, eczema	Decoction

Excoecaria acerifclia Fdidr.	Euphorbiaceae	jinni	Evergreen little ardor	Whole plant	Toothache, jaundice, cough	Decoction
Gaultheria yunnanensis Rehd.	Ericaceae	jiemaixiong	Evergreen shrub	Whole plant	Wound, toothache, eczema	Decoction (for internal using or for external using)
Gerbera anandria Sch.-Bip.	Compositae	baiyibeiding	Perennial herb	Root	Toothache, blood loss	Decoction with *Epaltes australis*
Girardinia palmata Gaud.	Urticaceae	ajiyan	Perennial herb	Whole plant	Edema, skin itch	Stewing with herb of maidenhair and pig's foot
Gynura segetum Merr.	Compositae	nianggemo	Perennial herb	Whole plant	Menstruation problems, wound, rheumatics	Decoction, squeeze juice (boiled water medicine)
Hedyotis pinifolia Wall.	Rubiaceae	niangjiebowo	Annual herb	Whole plant	Palmus, wound<br>New: ahypnia	Decoction with (without) buffalo's horn powder
Helicia erratica Hook. f.	Proteaceae	mitaile	Evergreen ardor	Root	Dyspepsia, enteritis, food poison, dysentery	Decoction with brown sugar
Hemerocallis plicata Stapf.	Liliaceae	guangyinshixing	Perennial herb	Root	Lung problems, stomachache	New: stewing with eggs
Houttuynia cordata Thunb.	Saururaceae	chaci'a	Perennial herb	Whole plant	Eczema, hemorrhoid;<br>New: hepatitis	Decoction with brown sugar
Hydrocotyle sibthorpioides Lam.	Umbelliferae	zebaiwa	Perennial creepy herb	Whole plant	Icteric hepatitis, cough, amygdalitis, eyes problems;	Decoction with (without) *Artemisia capillaris*
Hypericum patulum Thunb.	Hypercaceae	Yizhiqiaji yizhitaji	Half-evergreen shrub	Whole plant	Dog bites<br>New: snake bites	With root of *Vigna cylindrica* pounding into pulp for external using
Indigofera mengtseana Craib	Papilionaceae	gedugaduo	Sub-shrub	Root	Mastitis, joint pain, rheumatism, leprosy	Decoction with *Tetrastigma hypoglaucum*
Indigofera pseudotinctoria Mats.	Papilionaceae	kuchuxi	Sub-shrub	Whole plant	Amygdalitis, hemorrhoid, cold and cough	Decoction<br>New: stewing with mixed pork
Inula cappa DC.	Compositae	nitusai	Perennial herb	Whole plant	Chronic nephritis, hemorrhoid, cholecystitis	Decoction (for external using)
Knoxia valerianoides Thorel.	Rubiaceae	Shidao	Perennial herb	Root	Edema, hemophthisis;<br>New: ahypnia	New: soaking in alcohol

Lemma minor L.	Lemnaceae	yiwei	Hydrophytic little herb	Whole plant	Skin problems, edema	New: decoction with black bean
Leycesteria taiwanensis Wall.	Caprifoliaceae	panjiangtuo	Deciduous shrub	Whole plant	Cystitis, edema, hemorrhoid, hepatitis, fracture	New: stewing with hen and *Anemone cathayensis* roots
Litsea euosma W. W. Smith.	Lauraceae	shisuo	Deciduous little ardor	Whole plant	Dyspepsia, stomachache, wound	Decoction with fruits of *Crataegus pinnatifida*, pericarp of *Citrus madurensis*
Loxostigma griffithii Clarke.	Gesneriaceae	zuini	Perennial herb	Whole plant	Wound, dyspepsia, influenza	Decoction
Lycopodium complanatum L.	Lycopodiaceae	yizhea	Perennial herb	Whole plant	Fracture, wound<br>New: edema	Decoction or soaking in alcohol
Malva verticillata L.	Malvaceae	si'aobaobaop a	Perennial herb	Whole plant	Irregular menstruation; New: ahypnia, hematuria	New: stewing with pork
Marsdenia tenacissima Weight et Arm.	Asclepiadaceae	adazai	Deciduous ligneous liana	Whole plant	Bronchitis	Decoction
Munronia henryi Harms.	Meliaceae	lilutu	Dwarfish sub-shrub	Whole plant	Rheumatoid arthritis, stomachache, cold;	Decoction or soaking in alcohol
Murraya paniculata Jack.	Rutaceae	bennaxi	Evergreen shrub or little	Root, leaf	Hepatitis, stomachache, wound	New: making powder with *Zanthoxylum simulans* leaf
Paederia scandens Merr.	Rubiaceae	keqiliegu	Perennial herbal liana	Whole plant	Irregular menstruation	Decoction
Paris polyphylla var. *yunnanense* Hand.-Mazz.	Trilliaceae	niupalie	Perennial herb	Rhizome	Traumatism, wound, toothache, snake bite, arthritis, malaria	Decoction for oral-taking, grind powder for external or oral use
Patrinia scabiosaefolia Fisch ex Link.	Valerianaceae	sheweilong	Perennial herb	Whole plant	Schizophrenia, enteritis	New: decoction with *Lonicera japonica* flowers
Pholidota articulata Lindl.	Orchidaceae	feidoululie’er	Perennial adnascent	Whole plant	Headache, Cough, leucorrhea	Soaking in alcohol
Pholidota chinensis Lindl.	Orchidaceae	gushangye	Perennial herb	Whole plant	Wound, fracture	Making the formula or for external using
Photinia parvifolia Schneid.	Rosaceae	nianggenipa	Deciduous little ardor	Whole plant	Toothache, jaundice, wound	Decoction or making the formula for external using

Phryma leptostachya L.	Phrymataceae	zibai	Perennial herb	Whole plant	Eczema, wound, fracture	Decoction or stewing with pork
Pimpinella candolleana Wight et Arn.	Umbelliferae	yanruopame nhou	Perennial herb	Whole plant	Cough, cold, stomachache, dyspepsia	Decoction or powder, soaking in alcohol
Pinellia cordata N. E. Brown.	Araceae	beiga	Perennial herb	Tuber	Stomachache, wound;<br>New: headache	Making capsule covered with other medicine
Piper hancei Maxim.	Piperaceae	hannuonaci	Ligneous liana	Whole plant	Rheumatic arthritis, wound, toothache	soaking in alcohol
Pittosporum daphniphylloides Hayata.	Pittosporaceae	peimei	Ardor	Fruit, bark	Wound, dysentery, hypertension	Decoction
Pleione yunnanensis Rolfe.	Orchidaceae	zinuoqu	Perennial herb	Root	Cough, bacillary phthisis	New: making powder with honey
Plumbago zeylanica L.	Plumbaginaceae	yushu	Climbing sub-shrub	Root, leaf	Rheumatoid arthritis	Decoction or soaking in alcohol
Polygala arillata Buch.-Ham.	Polygalaceae	Yajie	Shrub	Root	Bacillary phthisis, lung problems, irregular menstruation, wound	Decoction or stewing with lean meat
Polygala tatarinowii Regel.	Polygalaceae	wuwumo	Annual herb	Root	Palmus, bronchitis, wound;<br>New: ahypnia	Decoction;<br>New: stewing with lean meat
Polygonum cymosum Trev.	Polygonaceae	eluomo	Perennial herb	Root	Diarrhea, stomachache, wound, dysentery, irregular menstruation	Decoction
Polygonum denticulatum Huang	Polygonaceae	nianyanqi	Perennial herbal liana	Root	Chronic hepatitis, dyspepsia, asthma, dysentery	Decoction or powder
Pyracantha fortuneana Li	Rosaceae	maizhesai	Shrub or ardor	Whole plant	Dysentery; New: leucorrhea, irregular menstruation	Making the formula
Quercus mongolica Fisch.	Fagaceae	bomiao	Deciduous ardor	Root, bark	Poisoning, dyspepsia, hemorrhoid, jaundice	New: Decoction with the roots and bark of *Populus bonatii*
Ranunculus ternatus Thunb.	Ranunculaceae	anaishi	Perennial herb	Root	Bacillary phthisis	Making medicated plaster
Reineckea carnea Kunth.	Liliaceae	tuosipian	Perennial evergreen	Whole plant	Rheumatic arthritis, asthma, wound, fracture, irregular	Decoction or stewing with lean meat

Rhodiola henryi Fu	Crassulaceae	haisainai	Perennial herb	Whole plant	Wound, fracture, laryngitis, dysentery	Soaking *Dactylicapnos scandens* in alcohol
Rubus ellipticus Smith var. *obcordatus* Focke.	Rosaceae	jiejiesairuo	Evergreen shrub	Root	Amypdalitis, toothache, dysentery, icteric hepatitis,	Decoction;<br>New: soaking in alcohol
Rubus xanthocarpus Bur. et Franch.	Rosaceae	chuxu	Sub-fruticose perennial herb	Whole plant	Dysentery	Decoction
Rumex nepalensis Spreng.	Polygonaceae	apeiaji	Perennial herb	Root	Constipation, parotitis, wound, dysentery, jaundice	New: decoction with (without) *Acanthopanax gracilistylus* bark
Salix babylonica L.	Salicaceae	yu'ela	Deciduous ardor	Branch, root must	Gonorrhea, infective hepatitis	Decoction with *Glycyrrhiza uralensis* rhizome
Schefflera venulosa Hams.	Araliaceae	peizuo	Evergreen little ardor	Whole plant	Stomachache, arthritis, wound, neuralgia	Decoction with (without) *Clematis* stems
Sedum multicaule Wall.	Crassulaceae	niupianwei	Fleshy herb	Whole plant	Laryngitis, wound, hypertension	Decoction, pounding into pulp for external using
Smilax mairei Lévl.	Liliaceae	tongduo nainengruo	Evergreen climbing	Root	Rheumatic arthritis, chronic gastritis, irregular menstruation,	New: decoction with *Lagopsis supina*
Solanum khasianum C. B Clarke.	Solanaceae	Taopaishenz e	Perennial herb	Whole plant	Stomachache, toothache, headache, wound, parotitis, cough	Decoction
Swertia patens Burkill.	Gentianaceae	luoruke	Perennial herb	Whole plant	Dyspepsia (kid), hepatitis	Decoction
Tetrastigma hypoglauca Cumpl.	Vitaceae	yuewuji	Twining herbal liana	Whole plant	Wound, bacillary phthisis	Decoction or soaking in alcohol
Toona sinensis Roem.	Meliaceae	genuze	Deciduous ardor	Root skin	Dysentery	Decoction or making formula
Tripterygium hypoglaucum Hutch.	Celastraceae	yigumeiban	Deciduous rampant shrub	Root skin	Eczema, wound;<br>New: bronchitis	Decoction<br>New: soaking in alcohol
